# *GmGRP-like* gene confers Al tolerance in *Arabidopsis*

**DOI:** 10.1038/s41598-018-31703-z

**Published:** 2018-09-11

**Authors:** Li Chen, Yupeng Cai, Xiujie Liu, Chen Guo, Weiwei Yao, Shi Sun, Cunxiang Wu, Bingjun Jiang, Tianfu Han, Wensheng Hou

**Affiliations:** 1 National Center for Transgenic Research in Plants, Institute of Crop Sciences, Chinese Academy of Agricultural Sciences, Beijing, 100081 China; 2 Ministry of Agriculture Key Laboratory of Soybean Biology (Beijing), Institute of Crop Sciences, Chinese Academy of Agricultural Sciences, Beijing, 100081 China

## Abstract

Aluminium (Al) toxicity restrains water and nutrient uptake and is toxic to plant roots, ultimately inhibiting crop production. Here, we isolated and characterized a soybean glycine-rich protein*-like* gene (*GmGRPL*) that is mainly expressed in the root and that is regulated by Al treatment. Overexpression of *GmGRPL* can alleviate Al-induced root growth inhibition in *Arabidopsis*. The levels of IAA and ethylene in *GmGRPL*-overexpressing hairy roots were lower than those in control and RNA interference-exposed *GmGRPL* hairy roots with or without Al stress, which were mainly regulated by *TAA1* and *ACO*, respectively. In transgenic soybean hairy roots, the MDA, H_2_O_2_ and O_2_^–·^content in *GmGRPL*-overexpressing hairy roots were less than that in control and RNA interference-exposed *GmGRPL* hairy roots under Al stress. In addition, IAA and ACC can enhance the expression level of the *GmGRPL* promoter with or without Al stress. These results indicated that *GmGRPL* can alleviate Al-induced root growth inhibition by regulating the level of IAA and ethylene and improving antioxidant activity.

## Introduction

Aluminium (Al) toxicity is a primary factor that reduces crop yields from acidic soils. In acidic soils (pH < 5), the phytotoxic Al^3+^ ion is dissolved from clay minerals and becomes soluble, producing Al toxicity that restrains water and nutrient uptake and is toxic to plant roots, ultimately inhibiting crop production. Approximately 30% of the world’s arable land and approximately 50% of the world’s potentially arable land are acidic soils^1–3^. Furthermore, up to 60% of the acidic soils in the world are in developing countries, where food production is critical^4,5^. Al toxicity has a more prominent impact on crop production and is also exceeded only by drought among the abiotic limitations to crop production.

The most dramatic symptom of Al toxicity is the inhibition of root elongation, which is caused mainly by the inhibition of cell expansibility and division^6^. As the inhibition of root elongation is observed within 30 min in Al-sensitive cultivar^7^, it is now generally accepted that Al inhibition of cell expansion is the main cause of the inhibition of root elongation^8^. Several reports have shown consistently that most Al is bound to the cell wall^9,10^. For instance, 85–90% of the total Al accumulated by barley roots is tightly bound to cell walls^11^. Almost 90% of the total Al is associated with the cell walls of cultured tobacco cells^9^. Removal of Al binding in apoplasts or cell walls by citrate restored root regrowth^12^. This illustrated that the amount of Al binding in the cell wall is an important factor in Al toxicity. Thus, improving Al tolerance may be achieved by adjusting the Al binding ability to the cell wall or modification of the cell wall components. Recent evidence has shown that the regulation of cell wall component-related genes can influence Al toxicity. Zhu *et al*. found that an *XTH31* T-DNA insertion mutant, *xth31*, is more Al resistant than the wild type. *xth31* accumulates significantly less Al in the root apex and cell wall, shows remarkably lower *in vivo* XET action and extractable XET activity, and has a lower xyloglucan content. This indicated that *XTH31* affected Al sensitivity by modulating the cell wall xyloglucan content and Al binding capacity^13^.

Glycine-rich proteins (GRPs) belong to the family of cell wall structure proteins and exhibit a diversity of structural domains, tissue-specific expression patterns, and functions. Additionally, this diversity led to the concept that GRPs should not be considered as a family of related proteins but as a wide group of proteins that share a common structural domain. It was indicated that GRPs likely participated in multiple physiological processes^14^. Many lines of evidence have shown that GRPs may improve plant tolerance by improving the stability and reconstruction of the cell wall. Overexpressing *AtGRP5* can enhance the elongation of the root and inflorescence axis in *Arabidopsis*. Thus, *AtGRP5* is likely involved in organ growth by promoting cell elongation^15^. *AtGRP9* is involved in lignin synthesis in response to salt stress in *Arabidopsis*^16^. *Arabidopsis AtGRP2* or *AtGRP7* could enhance seed germination and seedling growth under cold stress, and even confer freezing tolerance in *Arabidopsis*, as well as improve the grain yield of rice under drought stress^17–19^. A recent study reported that *AtGRP3* is implicated in root size and aluminium response pathways in *Arabidopsis*, and the *grp3-1* mutant exhibits longer root and Al tolerance^20^.

Here, we cloned a soybean glycine-rich protein*-like* gene (*GmGRPL*) that contains a glycine-rich protein domain; additionally, it was highly expressed in the root and was induced by Al. The root apex is the major target site of Al toxicity^21^. Thus, we investigated the role of *GmGRPL* in Al stress. We found that overexpression of *GmGRPL* can alleviate Al-induced root growth inhibition in *Arabidopsis*. In the soybean hairy roots system, we found that *GmGRPL* enhanced Al resistance by regulating the level of IAA and ethylene and improving the antioxidant activity.

## Results

### Isolation and bioinformatics analysis of *GmGRPL* and its promoter structure

**Figure 1:**
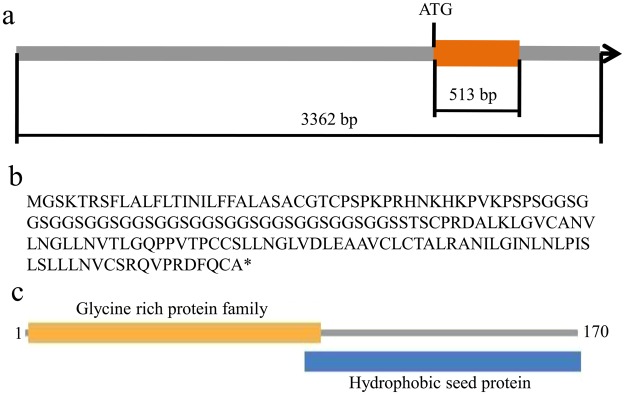
Diagram of the *GmGRPL* gene structure and protein domain. **(a**) *GmGRPL* gene structure. The *GmGRPL* cDNA is 3, 362 bp in length and contains an open reading frame (ORF) of 513 bp. (**b**) *GmGRPL* protein encodes 170 aa. (**c**) *GmGRPL* protein domain. The glycine-rich protein family domain is located in the N-terminus, and the hydrophobic seed protein domain is found in the C-terminus.

The *GmGRPL* gene is located on chromosome 17, with the gene number Glyma.17G139700. Its genomic sequence only contains one exon. The *GmGRPL* cDNA is 3, 362 bp in length and contains an open reading frame (ORF) of 513 bp that encodes a protein of 170 aa (Fig. 1a,b). The protein sequence analyses revealed a glycine-rich protein family domain and a hydrophobic seed protein domain in the GmGRPL protein. The glycine-rich protein family domain is located in the N-terminus, and the hydrophobic seed protein domain is found in the C-terminus (Fig. 1c).

A 1, 999-bp promoter fragment upstream of the ATG (start codon) was cloned, and the elements were examined using the PlantCARE web tool. Several putative cis-regulatory elements were deciphered from the promoter sequence of *GmGRPL* (Table S1). The TATA box sequence elements, which are required for critical and precise transcription initiation, existed in many forms in the promoter region. The CAAT BOX sequences were also found at numerous positions. Some hormone-response elements were present in the promoter region and included the ethylene-responsive element (ERE) and salicylic acid-responsive element (TCA). Some stress-response elements were also present, including heat stress-responsive elements (HSEs), drought stress-responsive elements (MBS), and many light-responsive elements (BOX 4, BOXI, GA, GT1, I-box, TCT).

### Expression pattern of *GmGRPL*

**Figure 2:**
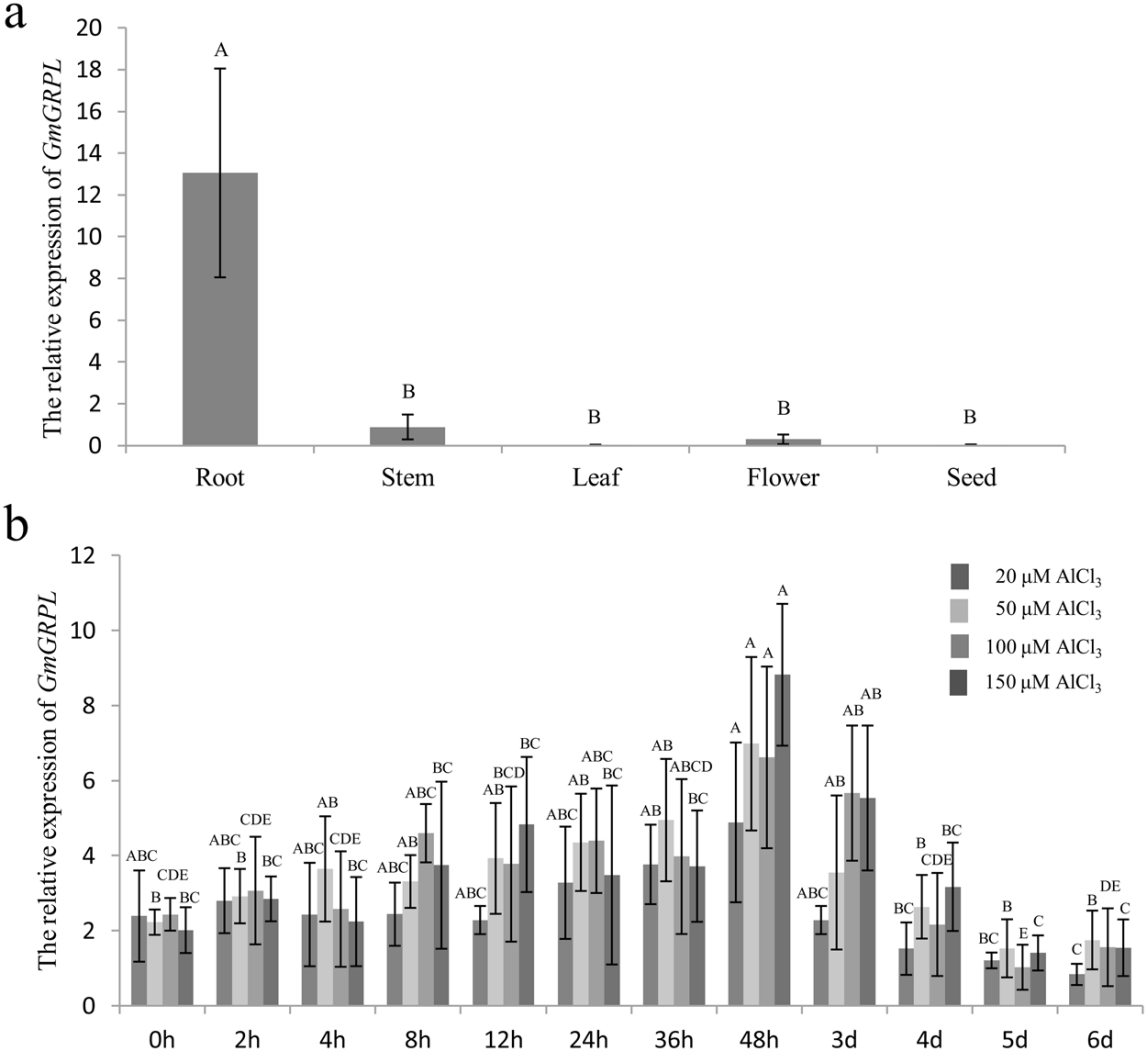
Tissue and Al-inducible expression pattern of *GmGRPL* by qRT-PCR. The soybean *GmActin* gene was used as the internal reference gene. (**a**) Tissue expression pattern of *GmGRPL* in root, stem, leaf, flower and seed. The different capital letters represent significant differences between tissues by ANOVA (*p* < 0.01). (**b**) Al-inducible expression pattern of *GmGRPL*. The soybean seedlings in the Vc stage (unifoliolate leaf fully developed) were transferred to AlCl_3_ (20, 50, 100, 150 μM) for 0, 2, 4, 8, 12, 24, 36, 48 h, 3 d, 4 d, 5 d and 6 d. The roots were sampled to analyse the Al-inducible expression pattern. The different capital letters represent significant differences during different times of one treatment by ANOVA (*p* < 0.01). The error bars represent the SEM. *n* = 3.

The *GmGRPL* tissue-specific expression pattern was evaluated by qRT-PCR at different developmental stages in the soybean cultivar ‘Williams 82’. The highest expression level of the *GmGRPL* transcript was detected in the root, and that in the stem and flower were relatively low; the expression in the leaf and seed were almost undetectable (Fig. 2a).

To determine the response of *GmGRPL* to Al stress, soybean seedlings in the Vc stage (unifoliolate leaf fully developed) subjected to Al treatments were used for qRT-PCR analysis. The expression of *GmGRPL* in roots was upregulated by Al treatments during 48 h, then it was gradually decreased after 3 days (Fig. 2b).

### Overexpression of *GmGRPL* in *Arabidopsis* promotes root growth and Al resistance

**Figure 3:**
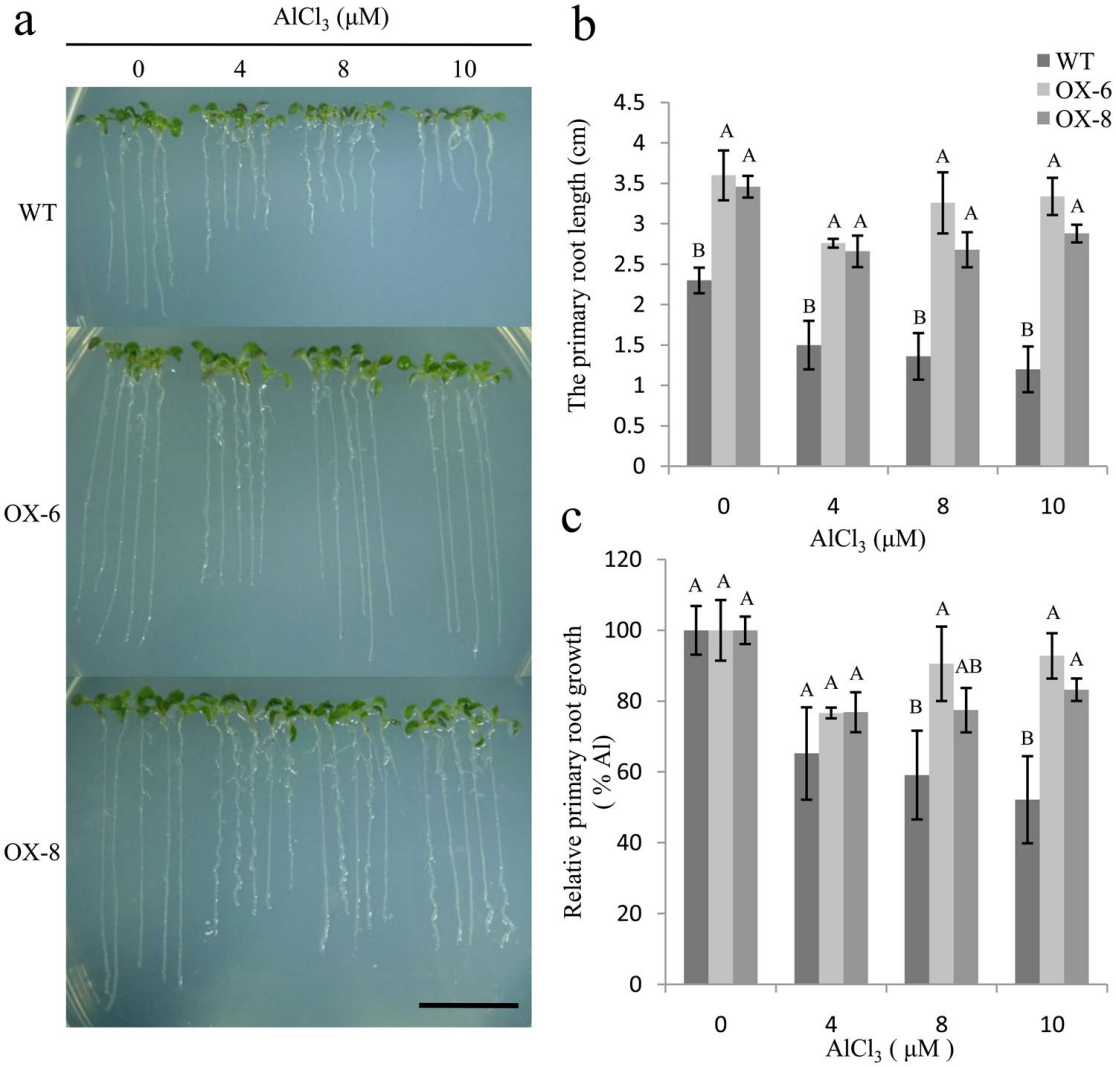
The root growth of wild type and *GmGRPL* overexpression *Arabidopsis* with or without Al treatment. The wild-type and overexpressing *GmGRPL Arabidopsis* seedlings grown in the 1/2 MS medium containing 0, 4, 8, or 10 µM AlCl_3_ (pH 4.5) for 9 d. (**a**) Phenotype of 9-day-old wild-type (WT) and *GmGRPL*-overexpression seedlings. OX-6 and OX-8 are transgenic lines. Bar, 1 cm. (**b**) The primary root length was measured. (**c**) The relative root length growth was calculated. The relative root length growth was calculated using the following formula: the root elongation under Al treatment/the root elongation in Al-free control ×100. The different capital letters represent significant differences between wild-type and *GmGRPL* overexpression lines by ANOVA (*p* < 0.01). The error bars represent the SEM. *n* = 5.

We investigated *GmGRPL* function by overexpressing *GmGRPL* in the *Arabidopsis* ecotype Col-0. *GmGRPL* promoted root growth compared with the wild-type plants. The primary roots in *GmGRPL*-overexpressing plants were longer than those in the wild-type (Fig. 3a,b). To further analyse the responses of root growth under Al stress, the *GmGRPL*-overexpressing *Arabidopsis* seedlings were grown on medium treated with different concentrations of AlCl_3_. Nine days after Al treatment, the root lengths were calculated. Root growth was inhibited in both the wild-type and transgenic lines, but the roots of the *GmGRPL*-overexpressing lines grew better than those of wild-type plants (Fig. 3a,b). The relative root growth of *GmGRPL*- overexpressing lines was also higher than that of wild-type plants (Fig. 3c).

### *GmGRPL* reduced IAA and ethylene level by regulating genes in IAA and ethylene biosynthetic pathway

**Figure 4:**
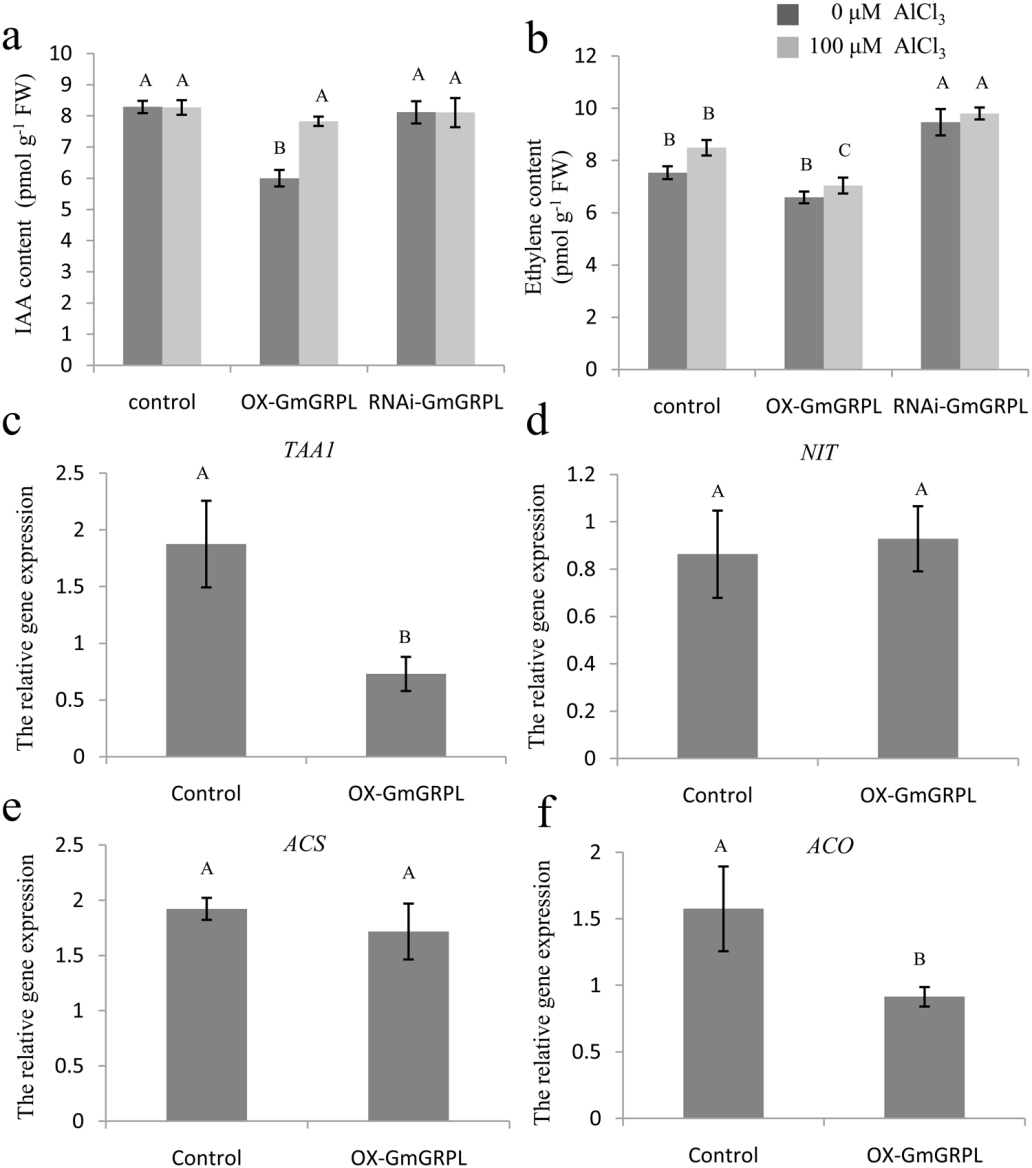
Changes in hormones of hairy roots under Al stress and the expression level of genes in its biosynthesis. 0.1 g hairy root tips (10 mm) were used to measure IAA and ethylene content by ELISA Kit. (**a**) IAA content. (**b**) Ethylene content. The different capital letters represent significant differences between treatments in control, *OX-GmGRPL* and *RNAi-GmGRPL* hairy roots by ANOVA (*p* < 0.01). (**c**–**f**) The expression level of *TAA1*, *NIT*, *ACS* and *ACO*. qRT-PCR analysis of gene expression levels between control and *OX-GmGRPL* hairy roots. *GmActin* was used as an internal reference. The different capital letters represent significant differences between control and *OX-GmGRPL* hairy roots by ANOVA (*p* < 0.01). The error bars represent the SEM. *n* = 3.

The indole-3-acetic (IAA) and ethylene are involved in the regulation of root growth. IAA and 1-aminocyclopropane-1-carboxylic acid (ACC) can inhibit root growth with or without Al in *Arabidopsis* and soybean (Figs S1 and S2). *GmGRPL*-overexpressing (*OX-GmGRPL*) hairy roots had lower IAA content (Fig. 4a). With 100 µM AlCl_3_ treatment, the IAA content was increased in *OX-GmGRPL* hairy roots, and there was no evident change in the IAA content in control and RNA interference-exposed *GmGRPL* (*RNAi-GmGRPL*) hairy roots, but the IAA level in *OX-GmGRPL* hairy roots was still lower than that in control and *RNAi-GmGRPL* hairy roots (Fig. 4a). *OX-GmGRPL* hairy roots also had lower ethylene content (Fig. 4b). With 100 µM AlCl_3_ treatment, the ethylene content was increased in control, *OX-GmGRPL* and *RNAi-GmGRPL* hairy roots. The ethylene level in *OX-GmGRPL* hairy roots was approximately 83% compared with that in control and 72% compared with that in *RNAi-GmGRPL* hairy roots (Fig. 4b).

Trp aminotransferase (TAA1) and nitrilase (NIT) are two key enzymes in IAA biosynthesis. In *OX-GmGRPL* hairy roots, the expression of *TAA1* was significantly reduced, and the expression of *NIT* was no obvious change (Fig. 4c,d). The IAA level in *OX-GmGRPL* hairy roots may be lowered by reducing the *TAA1*. ACC synthase (ACS) and ACC oxidase (ACO) are two key enzymes in ethylene biosynthesis. In *OX-GmGRPL* hairy roots, the expression of *ACS* was decreased slightly and the *ACO* was significantly reduced (Fig. 4e,f). The ethylene level in *OX-GmGRPL* hairy roots may be lowered by reducing *ACO*.

### *GmGRPL* improves antioxidant activity in soybean hairy roots

**Figure 5:**
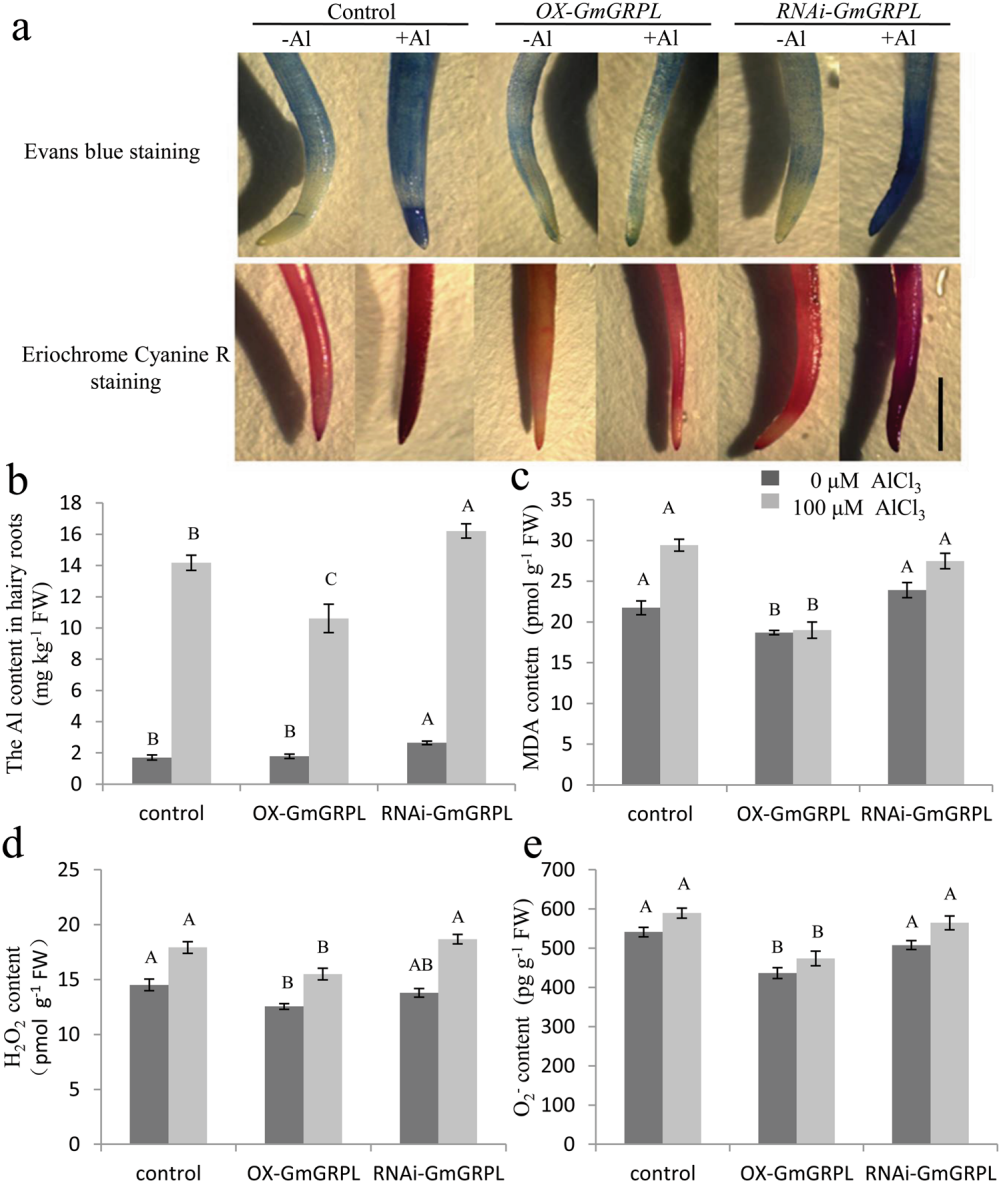
Changes in lipid peroxidation and antioxidant activity of hairy roots under Al stress. The control, *OX-GmGRPL* and *RNAi-GmGRPL* hairy roots were treated with or without 100 μM AlCl_3_ for 24 h. The hairy roots were excised for determination. (**a**) Evans blue staining and Eriochrome Cyanine R staining. After Al^3+^ treatment, the hairy roots were washed three times with sterilized water and were stained with Evans blue solution 0.025% (w/v) in 0.5 mM CaCl_2_ (pH 4.5) and Eriochrome Cyanine R solution 0.01% (w/v) for 10 min. The stained roots were washed in sterilized water until no dye elutes from the roots. The stained roots were photographed. Bar, 1 cm. (**b**) Al content in hairy roots. 0.5 g hairy roots were extracted to measure Al content using ICP-MS. (**c**) MDA content. (**d**) H_2_O_2_ content. (**e**) O_2_^−·^content. 0.1 g hairy root tips (10 mm) were homogenized with PBS buffer (pH 7.4), and the supernatant was used for reaction using ELISA Kits. The different capital letters represent significant differences between treatments in control, *OX-GmGRPL* and *RNAi-GmGRPL* hairy rootsby ANOVA (*p* < 0.01). The error bars represent the SEM. *n* = 3.

The transgenic-positive soybean hairy roots were used for Al treatment. The hairy roots induced by K599 strain carrying null vector were used as the control. With Al treatment, the hairy roots were stained with Evans blue and Eriochrome Cyanine R. Evans blue staining is used to detect membrane peroxidation and integrity. Eriochrome Cyanine R staining is an Al-dependent stain that can detect Al accumulation on the surface of the root. The results showed that Evans blue staining was enhanced in all hairy root types under Al stress, with stronger staining in control hairy roots than in *OX-GmGRPL* hairy roots and weaker staining in control hairy roots than in *RNAi-GmGRPL* hairy roots. The difference was more obviously observed in hairy root tips (Fig. 5a). *OX-GmGRPL* hairy roots displayed weaker Eriochrome Cyanine R staining than the control, while *RNAi-GmGRPL* hairy roots showed stronger staining than those of the control (Fig. 5a). Under Al stress, the Al content was increased in all hairy root types, but it was lower in *OX-GmGRPL* hairy roots than in control and *RNAi-GmGRPL* hairy roots (Fig. 5b). These results suggested that *GmGRPL* overexpression alleviates Al toxicity in the hairy roots.

Malondialdehyde (MDA) is the final product of lipid peroxidation, which indicates the extent of membrane peroxidation. Al treatment increased the MDA content in the control, *OX-GmGRPL* and *RNAi-GmGRPL* hairy roots. The MDA content was higher in control hairy roots than in *OX-GmGRPL* hairy roots and lower than that in *RNAi-GmGRPL* hairy roots (Fig. 5c). Similar results were shown with Evans blue staining. These results indicated that less lipid peroxidation was caused by Al stress in *OX-GmGRPL* hairy roots than in control hairy roots.

Al treatment also induced hydrogen peroxide (H_2_O_2_) and super oxygen anion-free radical (O_2_^−^) production in hairy roots after 24 h of treatment. The H_2_O_2_ content was increased by approximately 1.2-fold at 100 µM AlCl_3_ compared with that at 0 µM AlCl_3_ (Fig. 5d). The O_2_^−·^ content was increased by approximately 1.1-fold at 100 µM AlCl_3_ compared with that at 0 µM AlCl_3_ (Fig. 5e). Both the H_2_O_2_ and O_2_^−·^ content in *OX-GmGRPL* hairy roots was lower than that in control and *RNAi-GmGRPL* hairy roots.

### Histochemical detection of *GmGRPL* promoter in soybean hairy roots and *Arabidopsis*

**Figure 6:**
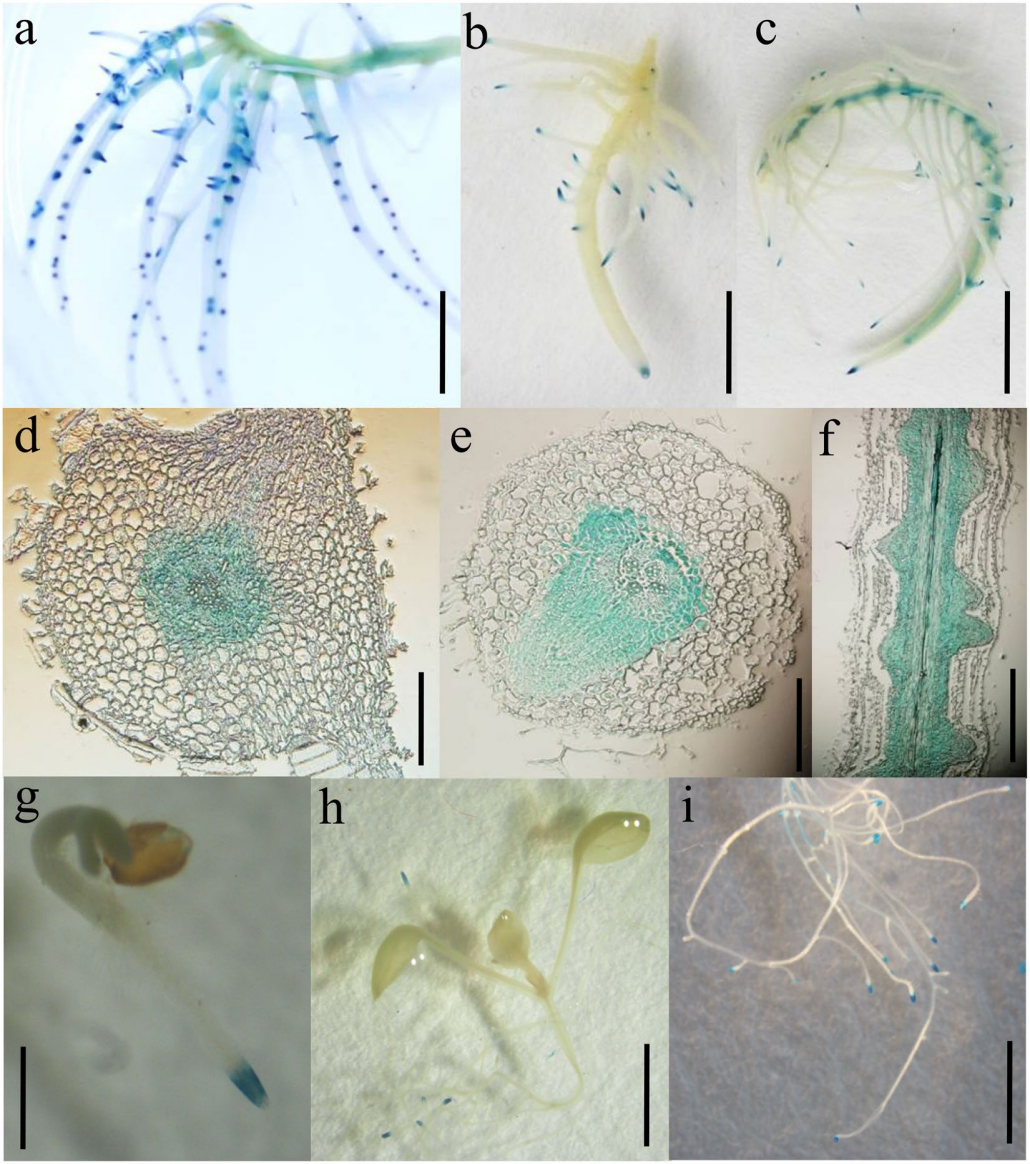
Expression pattern of the *GmGRPL* promoter. (**a**–**c**) GUS staining in soybean hairy roots (**a**,**b**) GUS staining only detect in hairy root tips (**c**) GUS staining detect in both root tips and stele. Bar, 1 cm. (**d**–**f**) Paraffin section for GUS-positive hairy roots (**d**,**e**) Cross section of GUS-positive hairy roots (**f**) Longitudinal section of GUS-positive hairy roots. Bar, 100 μm. (**g**–**i**) GUS staining detect only in root tips in transgenic *Arabidopsis* (**g**) Two-day-old seedling for GUS staining. Bar, 500 μm. (**h**) 10-day-old seedling for GUS staining. Bar, 1 mm. (**i**) The root of 20 d seedlings for GUS staining. Bar, 1 cm.

Transgenic *ProGmGRPL::GUS Arabidopsis* and soybean hairy roots were used for the analysis of the promoter expression pattern. GUS staining in hairy roots showed that the *GmGRPL* promoter was mainly expressed in hairy root tips (Fig. 6a,b); in some hairy roots, the *GmGRPL* promoter was detected in both the root tip and stele (Fig. 6c). The paraffin section also showed that the expression of *GmGRPL* promoter occurred in pericycle cells (Fig. 6d–f). GUS staining in transgenic *Arabidopsis* showed that the *GmGRPL* promoter was expressed only in the root tip, and GUS staining was not detected in other organs (Fig. 6g–i).

**Figure 7:**
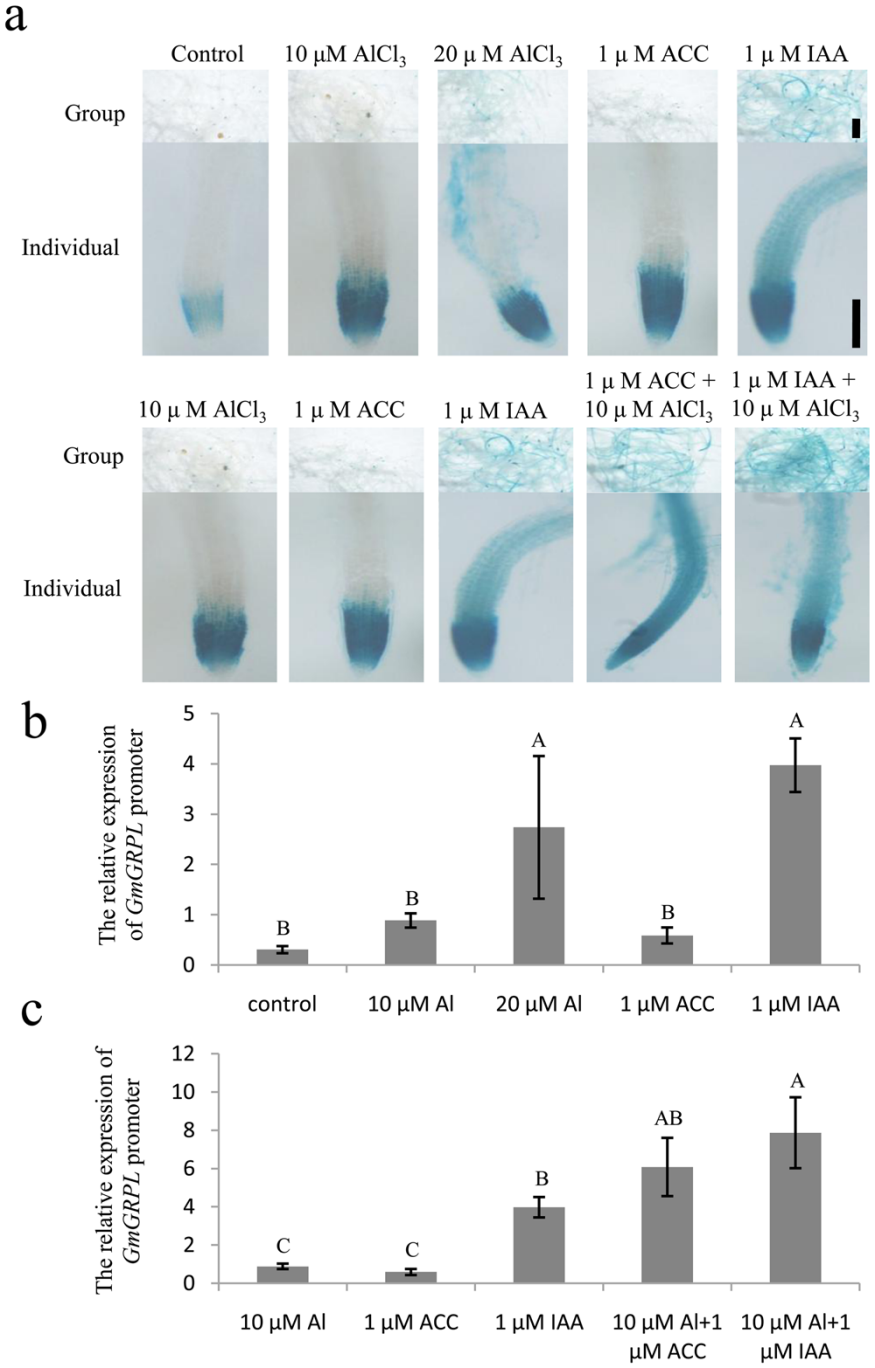
Inducible expression pattern of the *GmGRPL* promoter in *Arabidopsis*. Group represents 30 seedling roots together for GUS staining. Bar, 5 mm. Indivitual represents one root in the group. Bar, 100 μm. (**a**) GUS staining in transgenic *Arabidopsis* roots with single treatments of Al, ACC and IAA, and co-treatments of Al, ACC, and IAA. (**b**) Relative expression level of *GUS* with single treatments of Al, ACC and IAA in transgenic *Arabidopsis* roots. (**c**) Relative expression level of *GUS* with co-treatments of Al, ACC, and IAA in transgenic *Arabidopsis* roots. The different capital letters represent significant differences between treatments by ANOVA (*p* < 0.01). The error bars represent the SEM. *n* = 20.

To assess the inducible expression pattern of the *GmGRPL* promoter, the transgenic *ProGmGRPL::GUS Arabidopsis* seedlings were treated with hormones and Al stress. We tested the expression pattern in 10-day-old transgenic seedlings with 10 µM AlCl_3_, 20 µM AlCl_3_, 1 µM IAA, or 10 µM 1-aminocyclopropane-1-carboxylic acid (ACC) treatment for 24 h. In addition, after 10 µM AlCl_3_ treatment, the seedlings were also co-treated with 1 µM IAA and 10 µM ACC, respectively, for 24 h. The seedlings treated with H_2_O were used as the control. After treatment, the seedlings were subjected to GUS staining overnight. *GmGRPL* promoter activity was upregulated by AlCl_3_. Single IAA and ACC treatment could enhance the promoter expression. Furthermore, IAA and ACC increased the *GmGRPL* promoter expression under Al stress (Fig. 7a–c).

## Discussion

In this study, we described the novel gene *GmGRPL* which was located on chromosome 17, with the gene number Glyma.17G139700. The *GmGRPL* cDNA is 3, 362 bp in length and contains an ORF of 513 bp that encodes a protein with unknown function. The protein contained two protein domains, including a glycine-rich protein family domain and a hydrophobic seed protein domain (Fig. 1). We also obtained 1,999 bp promoter sequence of *GmGRPL*. Some hormone-related motifs were predicted to be present in the promoter region, including ethylene-responsive element (ERE) and salicylic acid-responsive element (TCA). The *GmGRPL* contains a typical glycine-rich protein domain which may be belonged to the family of cell wall structure proteins. Recent evidence has shown that the regulation of cell wall component-related genes can influence Al toxicity. In addition, the soybean *GmGRPL* gene is tissue specific and is highly expressed in the root (Fig. 2a), and the expression level of *GmGRPL* is regulated by Al (Fig. 2b). These findings suggest that *GmGRPL* may response to Al toxicity.

At present, several possible mechanisms for Al toxicity have been proposed; among them, organic acid secretion is considered the most important. Improving the secretion of malic acid, oxalic acid and citric acid, as well as other organic acids, can enhance aluminium tolerance^22–25^. For example, overexpressing *TaALMT1* enhanced malic acid secretion and Al resistance. A new interpretation about organic acid secretion has been proposed, indicating that the organic acids are more likely to interact with the cell wall, reducing the sites of Al and Al accumulation in the cell wall. The cell wall may play a more important role in Al resistance^26^. Yang reported that the ARF double mutant *arf10/16* showed greater Al resistance than the wild type. Many cell wall modification-related genes were regulated by *ARF10* and *ARF16*. The results showed that auxin-regulated Al-induced inhibition of root growth arose from auxin signalling-regulated modification of the cell wall structure or components. Cell wall modification is probably a further downstream response to Al and contributes to the auxin-mediated root growth inhibition in response to Al stress^27^. The upregulation of cell wall-regulated genes in the *arf10/16* double mutant primarily encoding cell wall structure proteins, including HRGPs, arabinogalactan proteins, and various Pro- and Gly-rich proteins, suggests that its reduced sensitivity to Al-induced inhibition of root growth is probably the outcome of a complex network, including structure assembly and remodelling. A potential role for HRGP may alter cell wall porosity and, as a result, reduces the mobility of Al in the root apoplast^28,29^. *XTH31* may alleviate Al toxicity by reducing the xyloglucan content of the cell wall, thereby lowering its Al binding sites^13^. Our study was performed using *Agrobacterium rhizogenes* and taking advantage of the soybean hairy root system. We can get the OX-hairy roots and RNAi-hairy roots *in vitro*, but the transgenic plants could not be generated by this system, the root growth test could not be achieved using hairy root system. And the root growth test with Al stress in our study was studied in transgenic *Arabidopsis*. Overexpressing *GmGRPL Arabidopsis* can reduce the inhibition of root growth under Al stress. The root length in transgenic *Arabidopsis* is longer than that in the control under Al stress (Fig. 3a,b). This finding suggests that the root shows less Al toxicity in overexpressing *GmGRPL Arabidopsis*. However, we found that the *GmGRPL* and *AtGRP3* have a different behaviour for Al stress. The *grp3-1* mutant exhibited longer root and Al tolerance^20^. According to the different structural domains and tissue-specific expression patterns of GRPs, we inferred that these diversities of GRPs may lead to different functions.

Auxin is the key regulator of root development^30–33^. Al-regulated inhibition of root growth is clearly regulated by auxin^34,35^. Auxin enhanced the Al-induced inhibition of root growth^27^. We found that *OX-GmGRPL* hairy roots showed increased IAA content with Al treatment. The *RNAi-GmGRPL* hairy roots showed similar levels of IAA as the control with or without Al treatment. However, the IAA level of *OX-GmGRPL* hairy roots with Al treatment was still lower than that of the control and *RNAi-GmGRPL* hairy roots (Fig. 4a). In addition, ethylene is involved in the regulation of root growth^27,36,37^. It has been reported that ethylene is important in Al-induced root growth inhibition in many species, including the common bean, *Arabidopsis* and wheat^22,35,38,39^. Ethylene can regulate auxin biosynthesis and basipetal auxin transport towards the elongation zone, activating auxin signalling in the root apex and causing root growth inhibition^40^. The results showed that, without Al treatment, the ethylene content in *OX-GmGRPL* hairy roots was lower than that in non-transgenic hairy roots and *RNAi-GmGRPL* hairy roots. Although ethylene was increased in all hairy roots types under Al treatment, the ethylene level of *OX-GmGRPL* hairy roots was still less than that of non-transgenic hairy roots and *RNAi-GmGRPL* hairy roots (Fig. 4b). We found that in *OX-GmGRPL* hairy roots, the expression of *TAA1* was significantly reduced, and the expression of *NIT* was no obvious change (Fig. 4c,d), and the expression of *ACS* was decreased a little and the *ACO* was significantly reduced (Fig. 4e,f). These results indicated that *GmGRPL* could reduce the IAA and ethylene levels by regulating *TAA1*and *ACO*. In addition, the promoter expression of *GmGRPL* was both upregulated by IAA and ACC (Fig. 7a). We inferred that *GmGRPL*, IAA and ethylene may interact on each other under Al stress.

Further study showed that the Al content is lower in *OX-GmGRPL* hairy roots than in the control under Al stress (Fig. 5b). The *OX-GmGRPL* hairy roots have higher ability of oxidative resistance than the control (Fig. 5c–e). Reactive oxygen species (ROS) are important for processes such as growth, development, and responses to biotic and abiotic environments. In plants, the root tips represent a zone of active ROS production^41^. Previous evidence has indicated that ROS affects the mechanical properties of cells, and an appropriate amount of ROS is essential for cell wall loosening^41^. However, a high level of ROS will cause cell wall stiffening and inhibition of cell expansion by inducing wall lignification^42^, as well as damage the integrity of the plasma membrane by causing lipid peroxidation^43^. We found that the MDA content is higher in control hairy roots than in *OX-GmGRPL* hairy roost (Fig. 5c). Furthermore, Evans blue staining in control hairy roots is stronger than that in *OX-GmGRPL* hairy roots (Fig. 5a). These results indicated that Al causes more serious oxidative stress in control hairy roots than in *OX-GmGRPL* hairy roots. The results showed that the levels of ROS, including H_2_O_2_, O_2_^−·^, in control hairy roots were higher than those in *OX-GmGRPL* hairy roots (Fig. 5d,e), suggesting that the Al-induced increase in ROS may be an important cause of Al-induced cell rigidity. Thus, *GmGRPL* also can enhance the antioxidant ability under Al stress.

In conclusion, this study revealed an important role of *GmGRPL* in Al resistance in *Arabidopsis*. The overexpression of *GmGRPL* was found to alleviate Al toxicity by regulating the level of IAA and ethylene and improving the antioxidant activity.

## Methods

### Plant materials and growth conditions

Soybean cultivar ‘Williams 82’ was cultivated in a greenhouse under 16 h light/8 h dark cycles at 28 °C. *Arabidopsis thaliana* ecotype Col-0 was planted at 22 °C under16 h light/8 h dark cycles.

### Isolation of *GmGRPL* and its promoter

Total RNA was isolated from ‘Williams 82’ roots using the TransZol Up Plus RNA Kit (TransGen Biotech). The primers for the amplification of *GmGRPL* cDNA were GmGRPL-F and GmGRPL-R (Table S2). The PCR product was purified and inserted into the pMD18-T cloning vector (Takara) and then sequenced. Genomic DNA was extracted using the CTAB method from ‘Williams 82’ leaves. According to the ‘Williams 82’ genomic data, an approximately 2-kb promoter sequence upstream from ATG was cloned and sequenced using specific primers (Pro-GmGRPL-F and Pro-GmGRPL-R) (Table S2). The *cis*-regulatory elements in the promoter were analysed by the PlantCARE database (bioformatics.psb.ugent.be/webtools/plantcare).

### Construction of the *ProGmGRPL::GUS* reporter plasmid and *OX-GmGRPL* and *RNAi-GmGRPL* plasmids

The plant expression vector pC13P1 has multiple cloning sites, a GUS reporter gene and no promoter. The pMD18-ProGmGRPL vector and pC13P1 vector were double digested with the restriction enzymes *Kpn*I and *Pst*I. The *GmGRPL* promoter was ligated into the pC13P1 vector with *Kpn*I and *Pst*I digestion to construct the *ProGmGRPL::GUS* vector. The selection vector pC(Delt)GUS, which has the hygromycin gene but not the GUS gene, was used for co-transformation. The *ProGmGRPL::GUS* vector and the selection vector pC(Delt)GUS plasmid were introduced into *Agrobacterium tumefaciens* GV3101 and *Agrobacterium rhizogenes* K599 using electroporation, respectively.

For the overexpression construct (*OX-GmGRPL*), the CDS of *GmGRPL* was inserted into the pGFPGUS vector with a CaMV 35S promoter, GFP and GUS reporter gene. The plasmid was introduced into *Agrobacterium rhizogenes* K599 and was used for transformation into soybean hairy roots. The CDS of *GmGRPL* was also inserted into a SP1300 vector with a super promoter and a hygromycin gene. The plasmid was introduced into *Agrobacterium tumefaciens* GV3101 and was used for transformation into *Arabidopsis*. For the RNAi construct (*RNAi-GmGRPL*), the CDS of *GmGRPL* was inserted into the vector pGFPGUS in the sense and antisense orientations. The plasmid was introduced into *Agrobacterium rhizogenes* K599 and was used for transformation into soybean hairy roots.

### *Arabidopsis* transformation and soybean hairy root transformation

The *Arabidopsis thaliana* ecotype Col-0 was used for transformation. The overexpression vector *super promoter::GmGRPL* was single transformed, and the *ProGmGRPL::GUS* vector and selection vector pC(Delt)GUS were co-transformed. The transformation was performed using the floral dip method. The seeds of T3 transgenic *Arabidopsis* were used in this study.

Five-day-old cotyledonary nodes were used for soybean hairy root production^44^. The expression vectors were *OX-GmGRPL* and *RNAi-GmGRPL*. Hairy roots were detected by GFP fluorescence microscopy (Nikon SMZ1500), and the positive hairy roots were used for Al treatment.

### Quantitative real-time PCR (qRT-PCR)

The soybean root, stem, leaf, flower, and seed were used to analyse the tissue expression pattern of *GmGRPL* by qRT-PCR. The soybean seedlings in the Vc stage (unifoliolate leaf fully developed) were transferred to AlCl_3_ (20, 50, 100, 150 μM) for 0, 2, 4, 8, 12, 24, 36, 48 h, 3 d, 4 d, 5 d and 6 d. The roots were sampled to analyse the Al-inducible expression pattern by qRT-PCR. *GmGRPL*-specific primers qPCR-GmGRPL-F and qPCR-GmGRPL-R were used for qRT-PCR. The soybean *GmActin* gene was used as the internal reference gene (Table S2). The *GUS* gene was used to analyse the inducible expression pattern of the *GmGRPL* promoter in transgenic *ProGmGRPL::GUS Arabidopsis* seedling roots. The *Arabidopsis AtActin* gene was used as the internal reference gene (Table S2). The *TAA1*, *NIT*, *ACS* and *ACO* genes were used to analyse the change of IAA and ethylene between control and *OX-GmGRPL* hairy roots by qRT-PCR (Table S2). qRT-PCR reactions were performed according to the three-step method using SYBR Green I dye and the ABI7500 instrument for qRT-PCR. For qRT-PCR, a total volume of 20 μL was used that contained 10 μL of SYBR Premix Ex Taq (2×), 0.4 μL of dye, 0.4 μL of 10 μM of the upstream or downstream primers, 2 μL of cDNA template, and 6.8 μL of ddH_2_O. Real-time PCR amplification using the standard three-step procedure for denaturation was performed as follows: 95 °C for 30 s, followed by 40 cycles of 95 °C for 5 s, and 60 °C for 30 s. The relative expression level was calculated using the comparative 2^−ΔΔCt^ method.

### Al^3+^ treatment and Al content measurement

Al^3+^ treatment was given in the form of aluminium chloride (AlCl_3_) prepared in 0.5 mM CaCl_2_ (pH 4.5). After treatment, the hairy roots were washed three times with deionized water. The hairy roots were cut and collected for Al measurement, respectively. Next, 0.5 g of tissue sample was placed in a tank containing 5 ml of HNO_3_ and 2 ml of H_2_O_2_ for 4 h and was digested in a microwave, followed by dilution to 25 g with water as the sample solution. The Al content was measured using Inductively Coupled Plasma Mass Spectrometry (ICP-MS) (Agilent 7700, USA).

### Root growth analysis

The overexpressing *GmGRPL Arabidopsis* seedlings were grown in the 1/2 MS medium containing 0, 4, 8, or 10 µM AlCl_3_ (pH 4.5) for 9 d. The wide-type *Arabidopsis* seedlings were grown in the 1/2 MS medium containing 0, 4, 8, 10 µM AlCl_3_ (pH 4.5) and 0, 10, 50 nM IAA or ACC for 9 d. The soybean ‘Williams 82’ seeds were grown in B5 medium containing 0, 10, 50, 100 µM AlCl_3_ (pH 4.5) and 0, 10, 50 nM IAA or ACC for 9 d. The seedlings were photographed and the root length was measured using a centimeter scale.

### GUS Staining

GUS staining was used to detect the *GmGRPL* promoter tissue and inducible expression pattern. Histochemical GUS assay was performed according to Jefferson^45^. The transgenic *Arabidopsis* and hairy roots with the *ProGmGRPL::GUS* vector were used to study the promoter expression pattern. The tissues were placed in GUS staining solution (50 mM sodium phosphate at pH 7.0, 0.5 mM potassium ferrocyanide, 0.5 mM potassium ferricyanide, 0.5 mg/ml 5-bromo-4 chloro-3-indolyl-β-D-glucuronide (X-Gluc), 0.1% Triton X-100 and 20% methanol) and were incubated at 37 °C overnight. After staining, the tissue samples were bleached with 50%, 70% and 90% ethanol for 1 h each and were immersed in 70% ethanol overnight. GUS staining was observed under a Nikon SMZ1500 microscope and was photographed with a Nikon DS-Fil.

### Evans blue and Eriochrome Cyanine R staining

Hairy roots were used for Al^3+^ treatment (0, 100 µM) for 24 h. After Al^3+^ treatment, the hairy roots were washed three times with sterilized water and were stained with Evans blue solution 0.025% (w/v) in 0.5 mM CaCl_2_ (pH 4.5) and Eriochrome Cyanine R solution 0.01% (w/v) for 10 min. The stained roots were washed in sterilized water until no dye elutes from the roots. The stained roots were photographed.

### Antioxidant activity measurement

Hairy root tip (10 mm) tissue samples (0.1 g each) were homogenized with Phosphate buffer saline (PBS) buffer (pH 7.4) at 8000 rpm and 4 °C for 30 min, and the supernatant was used for reaction. The H_2_O_2_ content was measured using an enzyme-linked immunosorbent assay (ELISA) kit (SU-B91025). The O_2_^−·^, MDA, IAA and ethylene contents were measured using ELISA kits (SU-B91178, SU-B91059, SU-B91094 and SU-B91090, respectively) according to the kit instructions.

## Electronic supplementary material

Supplementary information

## Data Availability

All data generated or analysed during this study are included in this published article (and its Supplementary Information files).
