# Supplementary material for: GmGRP-like gene confers Al tolerance in Arabidopsis
Source: Sci Rep. 2018 Sep 11;8:13601. doi: 10.1038/s41598-018-31703-z (PMC6134052; doi:10.1038/s41598-018-31703-z)
Supplement: Supplementary file 1 — Supplementary information [file 41598_2018_31703_MOESM1_ESM.pdf]

GmGRP-like gene confers Al tolerance in *Arabidopsis*

Li Chen[1, 2+], Yupeng Cai[1, 2+], Xiujie Liu[1, 2], Chen Guo[1, 2], Weiwei Yao[1, 2], Shi Sun[2], Cunxiang Wu[2], Bingjun Jiang[2], Tianfu Han[2], Wensheng Hou[1, 2*]

[1]National Center for Transgenic Research in Plants, Institute of Crop Sciences, Chinese Academy of Agricultural Sciences, Beijing, 100081, China.

[2]Ministry of Agriculture Key Laboratory of Soybean Biology (Beijing), Institute of Crop Sciences, Chinese Academy of Agricultural Sciences, Beijing, 100081, China.

Table S1 Putative *cis*-acting elements in the *GmGRPL* promoter by PlantCARE

Motif name	Sequence	Function
ACE	AAAACGTTTA	cis-acting element involved in light responsiveness
BOX 4	ATTAAT	part of a conserved DNA module involved in light responsiveness
BOX Ⅰ	TTTCAAA	light responsive element
CAAT-box	CAAAT/ CAAT	common cis-acting element in promoter and enhancer regions
ERE	ATTTCAAA	ethylene-responsive element
GA-motif	AAGGAAGA	part of a light responsive element
GT1-motif	GGTTAA	light responsive element
HSE	AAAAAATTTC	cis-acting element involved in heat stress responsiveness
I-box	CTCTTATGCT	part of a light responsive element
MBS	CAACTG	MYB binding site involved in drought-inducibility
TATA-box	TATA	core promoter element around -30 of transcription start
TCA-element	GAGAAGAATA	cis-acting element involved in salicylic acid responsiveness
TCT-motif	TCTTAC	part of a light responsive element

Table S2 The primers for cloning and real-time quantitative PCR

Primer	Sequence 5’-3’
GmGRPL-F	ATGGGTTCCAAAACTCGC
GmGRPL-R	TTAAGCACATTGGAAATC
Pro-GmGRPL-F	AATAGTAGGCTTCTCTAG
Pro-GmGRPL-R	CTTAAACTTTTTAGGG
qPCR-GmGRPL-F	ACGGGTTGTTGAACGTGACT
qPCR-GmGRPL-R	TGGGACTTGCCTTGAGCAAA
qPCR-GmActin-F	GAGCTATGAATTGCCTGATGG
qPCR-GmActin-R	CGTTTCATGAATTCCAGTAGC
qPCR-AtActin-F	AAGTCTTGTTCCAGCCCTCG
qPCR-AtActin-R	TTTGCTCATACGGTCAGCGA
qPCR-GUS-F	TACCGACGAAAACGGCAAGA
qPCR-GUS-R	CGGTGATATCGTCCACCCAG
qPCR-TAA1-F	TCTCTCGCCTTGAACGTCAG
qPCR-TAA1-R	TGCTGCTAGCTCCTGTTGTG
qPCR-NIT-F	TTGTGTGTCAGTGGGTGTGAA
qPCR-NIT-R	ATGAGACGAGAGCAAGTGGC
qPCR-ACS-F	TGAGTCCACTGTTGGAGAAGC
qPCR-ACS-R	TAGTCTCTCCAGTGCCACTCCC
qPCR-ACO-F	AGGAAGGCTACCCTTAACCAGA
qPCR-ACO-R	GCAAGAAGAACGTGCTTTCCCA

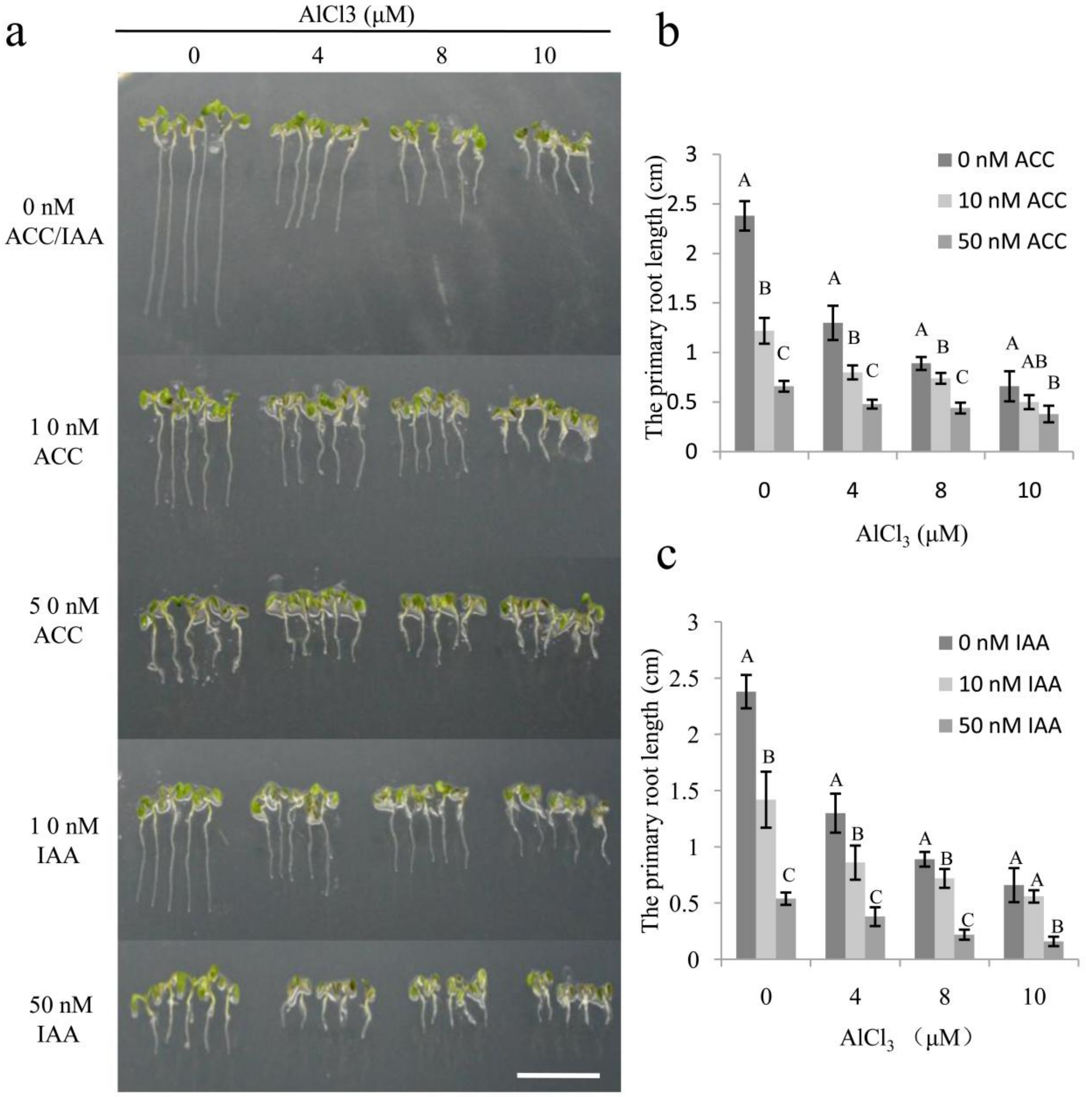

Fig. S1 The IAA and ACC regulate root growth with Al treatment in *Arabidopsis*. The wide-type *Arabidopsis* seedlings were grown in the 1/2 MS medium containging 0, 4, 8, 10 μM $AlCl_3$ (pH 4.5) and 0, 10, 50 nM IAA or ACC for 9 d. The seedlings were photographed and the root length was measured using a centimeter scale. (a) Phenotype of 9-day-old wide-type *Arabidopsis* seedlings with ACC or IAA under Al stress. Bar, 1cm. (b) The primary root length was measured only with ACC and Al. (c) The primary root length was measured only with IAA and Al. The different capital letters represent significant differences between treatments by ANOVA ($p < 0.01$). The error bars represent the SEM. $n = 5$.

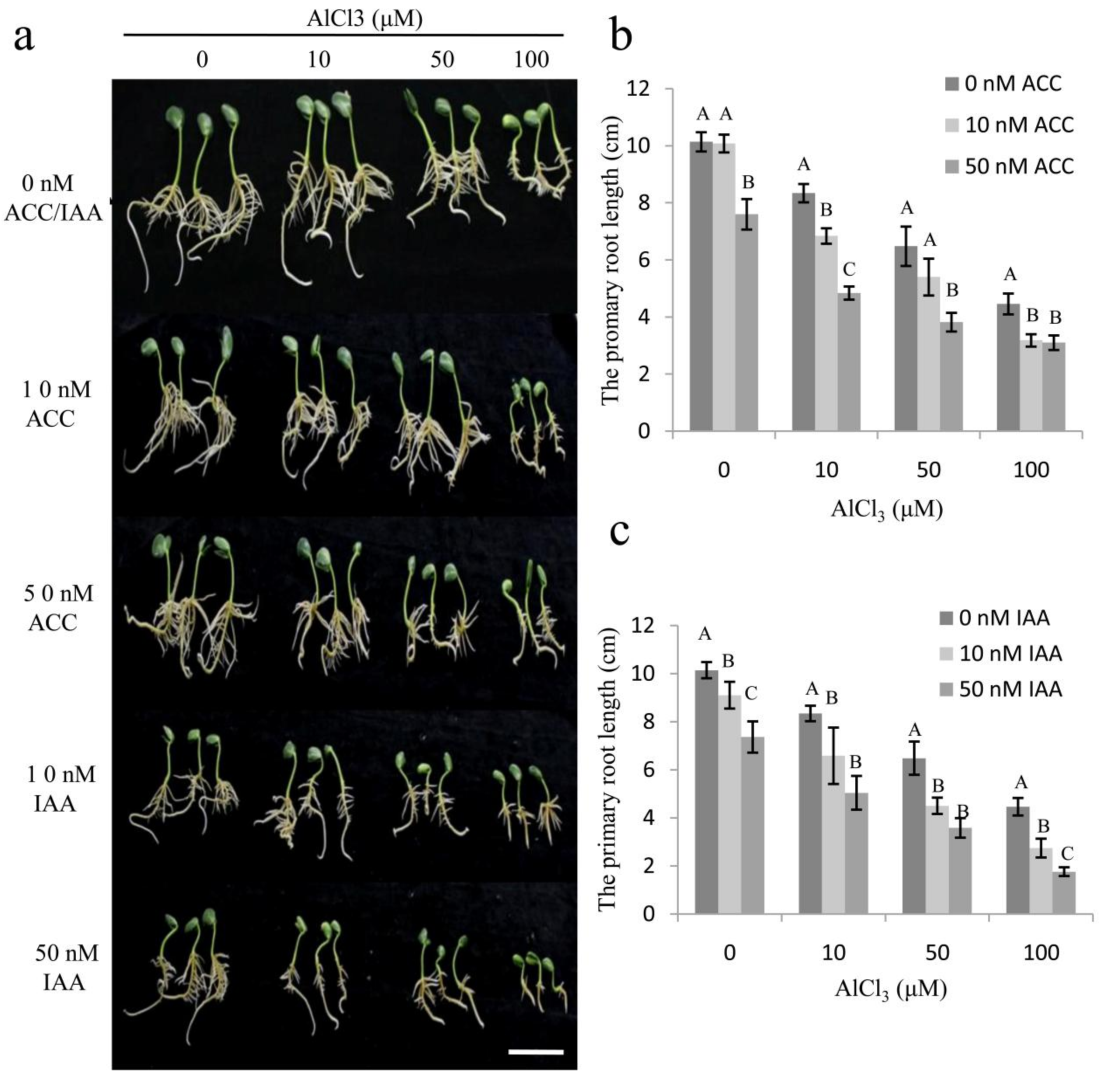

Fig. S2 The IAA and ACC regulate root growth with Al treatment in soybean. The soybean 'Williams 82' seeds were grown in B5 medium containing 0, 10, 50, 100 μM $AlCl_3$ (pH 4.5) and 0, 10, 50 nM IAA or ACC for 9 d. The seedlings were photographed and the root length was measured using a centimeter scale. (a) Phenotype of 9-day-old seedlings with ACC or IAA under Al stress. Bar, 5 cm. (b) The primary root length was measured only with ACC and Al. (c) The primary root length was measured only with IAA and Al. The different capital letters represent significant differences between treatments by ANOVA ($p < 0.01$). The error bars represent the SEM. $n = 5$.
